# Does Physical Fitness Buffer the Relationship between Psychosocial Stress, Retinal Vessel Diameters, and Blood Pressure among Primary Schoolchildren?

**DOI:** 10.1155/2016/6340431

**Published:** 2016-10-04

**Authors:** Markus Gerber, Katharina Endes, Christian Herrmann, Flora Colledge, Serge Brand, Lars Donath, Oliver Faude, Uwe Pühse, Henner Hanssen, Lukas Zahner

**Affiliations:** ^1^Department of Sport, Exercise and Health, University of Basel, Basel, Switzerland; ^2^Psychiatric Clinics of the University of Basel, Center for Affective, Stress and Sleep Disorders, Basel, Switzerland

## Abstract

*Background*. Strong evidence exists showing that psychosocial stress plays an important part in the development of cardiovascular diseases. Because physical inactivity is associated with less favourable retinal vessel diameter and blood pressure profiles, this study explores whether physical fitness is able to buffer the negative effects of psychosocial stress on retinal vessel diameters and blood pressure in young children.* Methods*. 325 primary schoolchildren (51% girls, M_age_ = 7.28 years) took part in this cross-sectional research project. Retinal arteriolar diameters, retinal venular diameters, arteriolar to venular ratio, and systolic and diastolic blood pressure were assessed in all children. Interactions terms between physical fitness (performance in the 20 m shuttle run test) and four indicators of psychosocial stress (parental reports of critical life events, family, peer and school stress) were tested in a series of hierarchical regression analyses.* Results*. Critical life events and family, peer, and school-related stress were only weakly associated with retinal vessel diameters and blood pressure. No support was found for a stress-buffering effect of physical fitness.* Conclusion*. More research is needed with different age groups to find out if and from what age physical fitness can protect against arteriolar vessel narrowing and the occurrence of other cardiovascular disease risk factors.

## 1. Introduction

Cardiovascular diseases are the most frequent cause of premature mortality in developed countries [1]. The American Heart Association states that ideal cardiovascular health consists of three biomarkers of cardiometabolic health (low blood pressure, cholesterol, and fasting glucose levels) and four health behaviours (non-smoking, body mass index [BMI] < 25 kg/m^2^, healthy eating, and regular physical activity) [2]. Concerning this last behaviour, the WHO ranks physical inactivity as the fourth most important global risk factor for future mortality [3]. In addition, strong epidemiological evidence exists showing that psychosocial stress plays an important part in the development of cardiovascular diseases [4].

In a conceptual framework developed by Hamer [5], pathophysiological pathways which may link psychosocial stress with cardiovascular diseases are proposed. Specifically, it is suggested that psychosocial stress might impact psychobiological processes, including hypothalamic-pituitary-adrenal and sympathetic nervous system function, endothelial dysfunction, inflammation, and autonomic control. It is further hypothesised that psychosocial stress has a negative impact on health behaviours (e.g., curbing motivation for physical activity), which in turn impairs the aforementioned biological processes, hereby increasing the risk of developing risk factors for or a manifest cardiovascular disease. Various protective factors (e.g., cardiorespiratory fitness) may influence these relationships in favourable ways and thus modulate the detrimental effects associated with high personal stress [6]. In support of this framework, using a gender-matched stratified sample, a study with Swedish adults showed that participants with high psychosocial stress who also had high cardiorespiratory fitness had lower systolic and diastolic blood pressure, low density lipoprotein cholesterol, triglycerides, and total cardiometabolic risk than participants with high stress, but low or moderate cardiorespiratory fitness levels [7]. Based on these findings, the authors concluded that higher cardiorespiratory fitness may provide some protection against the health hazards associated with high chronic stress by attenuating the stress-related increase in cardiovascular disease risk factors.

Further corroborating these results, research in older children and adolescents has shown that higher levels of physical fitness and regular physical activity have the potential to protect against the negative consequences of stressful life circumstances [8]. Brown and Lawton [9] demonstrated that stressful life events were negatively associated with self-reported health among children with infrequent exercise habits, but not among regular exercisers. Similar results were reported in a 9-month longitudinal study with 344 females in grades 7 through 11 by showing that the negative impact of stressful life events on health increased as exercise levels decreased [10]. Among 16-year old adolescents, Norris et al. [11] found that the negative relationship between perceived psychological stress and psychological well-being (depression and anxiety) was attenuated among students who had participated in 10 weeks of high intensity exercise training (at 70–75% of maximum heart rate), whereas no such moderation occurred in controls. Haugland et al. [12] demonstrated with a sample of 1670 students aged 11 to 16 years that high perceived school-related stress was related to significantly more psychosomatic complaints among students with low levels of self-reported leisure time physical activity, compared to their more active peers. However, this finding could not be replicated in a sample of Swiss students [13]. Furthermore, Sigfusdottir et al. [14] showed with a representative sample of 7232 Icelandic adolescents that, among girls who experienced high family conflict, those with high physical activity levels reported lower levels of depressive symptoms than their less active peers. Finally, a study with 303 American youth yielded the result that physical activity and perceived stress interacted in the prediction of BMI, sum of skinfolds, and waist circumference, showing that regular physical activity acts as a protective factor against the effects of chronic stress on obesity-related outcomes [15].

In summary, the majority of previous investigation with young people corroborates the hypothesis that regular exercise participation has the potential to protect against the health hazards associated with high psychosocial stress perceptions [16]. Nevertheless, it is apparent that prior studies have almost exclusively focused on older children and adolescents and used subjective health indicators, whereas research with younger children and objectively assessed cardiovascular disease risk factors is missing. In our opinion, this dearth is surprising for two reasons: First, cardiovascular risk factors are established early in life and track into adulthood [17, 18]. Second, previous research has shown that higher average daily moderate-to-vigorous physical activity is associated with a reduced cardiovascular risk among both European and American children [19, 20].

Lately, researchers have suggested that alterations of retinal vessel diameters are associated with an unfavourable cardiovascular risk profile in both adults and children. Narrower arteriolar and wider venular diameters are considered biomarkers for increased cardiovascular risk [21]. In adult populations, previous research has shown that altered retinal vessel diameters are associated with increased risk of obesity [22], hypertension [23], stroke [24], and cardiovascular mortality [25]. A number of studies show a clear gradient between narrower retinal arterioles and increased blood pressure [26, 27]. Relative to research with adult populations, studies with children are scarce. In recent years, however, we have been among a number of research groups, which have started to examine the relationship between retinal vessel diameter and blood pressure and body composition in children. The existing studies reveal that narrower retinal arteriolar diameters (CRAE), wider retinal venular diameters (CRVE), and a lower arteriolar to venular ratio (AVR) are associated with increased body mass index (BMI) and obesity-related risk factors [27–32]. Significant associations were also found for CRVE with height, weight, and waist circumference [27, 28]. Likewise, arteriolar narrowing and venular dilatation in young children were associated with higher blood pressure [30, 31]. These studies further show that among preschool and primary school children, retinal arteriolar narrowing and/or wider venular diameters are associated with higher systolic and diastolic blood pressure and increased BMI [30, 33]. So far, however, research on the relationship between psychosocial stress and retinal vessel diameters is missing, both in children and in adults.

Given the global disease burden associated with cardiovascular diseases [34] in combination with the fact that physical inactivity is related to less favourable retinal vessel diameter profiles both in adults and in children [27, 35–37], such insights seem urgently warranted. For instance, wider retinal venular diameters were found in physically active seniors compared to sedentary controls [35], whereas AVR proved to be positively related to fitness levels (anaerobic threshold) and improved after 10 weeks of exercise training [37]. Similar relationships were found in children. Thus, in a German sample of 578 children (aged 10–13 years) physically inactive children had lower arteriolar to venular ratio, which was mainly attributable to wider venular diameters [27]. Likewise, in a study with 1492 Australian children (aged 6 years), time spent in outdoor sporting activities was associated with wider arteriolar diameters [36]. Since alteration of retinal vessel diameters seems to manifest before common cardiovascular risk factors become evident [28], using retinal vessel diameters as outcomes in children seems particularly justified. Hence, the intention of the present study was to expand previous research by examining the role of physical fitness as a stress-buffer (i) in a sample of 6- to 8-year-old primary schoolchildren and (ii) with retinal vessel diameters and blood pressure as outcomes.

## 2. Materials and Methods

### 2.1. Study Design

As a part of a large scale, cross-sectional research project, all first grade pupils of public primary schools in the canton Basel-Stadt, Switzerland, took part in the Sportcheck study in 2014. This study was designed to monitor physical fitness, body composition, retinal vessel diameters, and blood pressure in all first grade primary schoolchildren. Ethical approval was obtained by the ethics committee of the University of Basel (EKNZ, Basel, number 258/12), and all study participants and their families provided written informed consent. The study procedures were carried out in accordance with the Declaration of Helsinki (1964).

### 2.2. Participants

Weight and height were assessed in 1255 children. Within this sample, parental/guardian consent was available for 540 children (43%) to participate in additional tests investigating physical fitness, psychosocial stress and well-being, blood pressure, and retinal vessel diameters. As 149 children dropped out due to relocation or illness at one of the two test dates, the final sample of children who participated in the fitness test, the anthropometric, blood pressure, and retinal microvascular measurements was *n* = 391. None of the children included in the analysis had chronic or ophthalmic disease or took medication or supplementation. Teachers and parents were informed about the study and its objectives prior to the children's participation. The parents provided information about the children's psychosocial stress. Complete data sets were available for 325 children (165 girls, 160 boys, M_age_ = 7.28 years, SD = 0.36).

### 2.3. Measures

#### 2.3.1. Static Retinal Vessel Analysis

The Static Retinal Vessel Analyzer (SVA-T, Impedos Systems UG, Jena, Germany) was used to assess children's retinal vessel diameters, which is a noninvasive and nonmydriatic online method of retinal vessel diameter assessment. The system consists of a Topcon fundus camera and an advanced image processing unit (Visualis 2.80, Visualis, Imedos Systems UG, Jena, Germany) [26]. A detailed method description is provided by Hanssen et al. [27]. In order to calculate central retinal arteriolar (CRAE) and venular (CRVE) equivalents based on the Parr-Hubbard formula [26], four valid digital images were taken from the retina of the left and the right eye, using an angle of 30° with the optic disc in the center [38]. All retinal arterioles and venules in the outer ring-zone of the automatic software were differentiated by a single experienced examiner. The AVR was calculated based on CRAE and CRVE. Measuring units (mu) are used to report retinal vessel diameters, with one mu corresponding to 1 *μ*m in the model of Gullstrand's normal eye. A single experienced examiner performed the assessment of the retinal vessels. This procedure resulted in satisfactory reproducibility, with correlation coefficients being high for all three parameters (CRAE: *r* = 0.94, coefficient of variation [CV] = 2.1, *p* < 0.001 each; CRVE: *r* = 0.95, CV = 2.3, *p* < 0.001 each).

#### 2.3.2. Blood Pressure

After a rest period of five minutes, blood pressure was measured on the bare right arm, following the recommendations of the American Heart Association [39]. The assessment took place in a comfortable position in a quiet room. An automated oszillograph (Oscillomate, CAS Medical Systems, Branford, CT, USA) was used to reduce interobserver variability. The measurements were repeated five times and the three values with the smallest variation were used for further analyses [40].

#### 2.3.3. Physical Fitness

Children's fitness was assessed during a physical education lesson in a group setting with the 20 m shuttle run test. Before the testing, a 5 min standardized warm-up was performed with all children. The 20 m shuttle run test provides an estimate of children's endurance performance. The reliability and validity of this test have been established previously [41, 42]. Moreover, the 20 m shuttle run test was proven to be sensitive to change in previous research [43]. During the test, the children run back and forth for 20 m, with initial running speed (8.0 km/h) being increased by 0.5 km/h every minute, paced by beeps on a stereo. If a child was unable to cross the 20 m line at the moment of the beep for two successive 20 m runs, the individual maximum was reached and the test ended. The number of stages completed, with one stage corresponding to one minute, were counted with a precision of 0.5 stages [44].

#### 2.3.4. Body Mass Index

A wall-mounted stadiometer was used to measure body height, without shoes and to the nearest 0.2 cm (Seca 206, Seca, Basel, Switzerland), while an electronic scale was used to assess body weight (Seca 899, Seca, Basel, Switzerland), in light clothing and without shoes and to the nearest 50 g. Body mass index (BMI) was obtained by dividing body weight in kg by height in meters squared.

#### 2.3.5. Parental/Guardian Questionnaire

Parents/guardians completed a questionnaire to assess parental/guardian education, sex, and age of their children. Parental/guardian educational level was operationalized with the highest completed school level. In addition, parents/guardians were asked to provide information about the level of psychosocial stress experienced by their children.

Parents/guardians completed two separate instruments to report their children's stress levels. To assess recent critical life events, parents/guardians filled in a 16-item adapted version of the Life Events Checklist (LEC) by Johnson and McCutcheon [45]. Examples of life events affecting oneself or significant others are death of a loved one, illness or accident of a loved one, persistent quarrel with brothers/sisters, divorce/separation of parents, and father/mother losing job. This list is not exhaustive but provides a representative sample of significant life events common during childhood [46]. Previous studies have shown that this instrument, which is widely used in child stress research [47, 48], has satisfactory reliability and validity [49]. The prevalence of the 16 life events was collected for the past three months. Moreover, parents rated the impact of each event on their children's life, using a 4-point scale from 0 (no impact) to 3 (large negative impact). The mean influence of all events was calculated as a trauma-indicator. Thus, children with few life events with a strong influence could have higher scores compared to subjects with several but moderate life events.

In addition, three subscales (family, friends, and school) of the KINDL-R questionnaire [50] were used to assess specific sources of stress in the lives of the participating schoolchildren. Parents were asked to respond on a 5-point Likert scale ranging from 0 (never) to 4 (all the time). All items start with the anchor: “During the past week …” The subscales used in this study were family (e.g., “my child got on well with us as parents,” “we quarrelled at home”), friends (e.g., “my child got along well with his friends”, “my child felt different from other children”), and everyday functioning at school (e.g., “my child easily coped with schoolwork”, “my child worried about his/her future”). The KINDL-R subscales have a high degree of reliability and satisfactory convergent validity. Moreover, the acceptance of the measure is high among both children and parents [50, 51]. Positively poled items were recoded before computing the subscale mean scores, so that higher scores reflected increased psychosocial stress throughout all dimensions.

### 2.4. Statistical Analyses

Sample size was estimated based on a prior investigation in physical fitness and retinal vessels in adults [37]. In this study, a strong association was found between cardiorespiratory fitness and retinal vessel diameters (Cohen's* d* = 1.2). Because effect size for the association of a 20 m shuttle run performance and retinal vessel diameters needs to be considered as smaller, we hypothesised a moderate effect size. Therefore, the estimated sample size was approximately 290 children in total (using G-Power software for* F*-tests; *F* = 0.25, power = 0.90, 5% level of significance).

Descriptive statistics (M, SD) were calculated to describe the baseline characteristics of the sample. Univariate analyses of variance (ANOVAs) and Pearson product moment correlations were run to examine how social and demographic factors were related to the study variables. Pearson product moment correlation coefficients were also used to examine bivariate associations between the predictor, moderator, and outcome variables. Correlations of *r* < 0.30 were considered small, with *r* = 0.30 to 0.50 as medium and *r* > 0.50 as large [52]. Thereafter, hierarchical (four-stage) regression analyses were performed to determine whether psychosocial stress interacted with physical fitness in the prediction of blood pressure and retinal vessel diameters. In sum, 20 separate regression equations were computed to test the influence of the four distinct stress indicators (critical life events, family, peer, and school stress) and the moderator variable (physical fitness) on all five outcomes (CRAE, CRVE, AVR, SBP, and DBP). To control for demographic and social background, sex (female = 1, male = 0), age, and parental/guardian education were entered in the first step in each regression. Moreover, BMI was controlled in the first step. Stress was then entered in the second step, physical fitness in the third step, and the interaction term between stress and fitness in the fourth step [53]. To avoid problems associated with multicollinearity, stress and fitness were centered (by computing* z*-standardized scores) before the interaction terms were calculated. Based on the guidelines of the American Psychological Association [54], the following coefficients are displayed in the results section: (i) the multiple correlation coefficient squared *R* ^2^ for the whole model after the final step, (ii) stepwise changes in *R* ^2^ (Δ*R* ^2^), and (iii) the standardized regression weights (*β*) for each predictor variable (for the final model). In order to consider refractive error, which might vary quite a lot in children around 7 years old, we included CRVE as a covariate in the models with CRAE as the outcome and CRAE in the models with CRVE as the outcome. In case of significant interaction terms, interaction effects were plotted using procedures as proposed by Dawson [55]. Alpha was set at *p* < 0.05 across all analyses. All statistical analyses were carried out using SPSS 23 for Mac (IBM, Armonk, New York, USA).

## 3. Results

### 3.1. Descriptive Statistics and Sociodemographic Influences

Table 1 provides a summary of the descriptive statistics for all predictor, moderator, and outcome variables. The mean for the critical life events trauma-indicator was relatively low (M = 1.54, SD = 2.58) with values ranging from 0 to 17 (possible range: 0–48). Likewise, relatively low scores were found for family stress (M = 0.70, SD = 0.47, actual range: 0–2, possible range: 0–4), peer stress (M = 0.80, SD = 0.50, actual range: 0–3, possible range: 0–4), and school stress (M = 0.42, SD = 0.42, actual range: 0–2.25, possible range: 0–4). The mean scores for SBP (M = 104.62 mmHg, SD = 8.03; range: 82.33–129.67) and DBP (M = 65.66 mmHg, SD = 6.87; range: 45.33–84.33) were comparable to those for children in the same age found in the German Health Interview and Examination Survey for Children and Adolescents (KiGGS 2003–2006) [56]. Likewise, the mean CRAE (M = 205.13 mu, SD = 13.85, range: 153.23–238.15), CRVE (M = 231.28 mu, SD = 13.03, range: 202.10–276.95), and AVR (M = 0.89, SD = 0.05, range: 0.68–1.02) were comparable to those found in a previous study with German children [27].

Differences between boys and girls were found with regard to peer stress (boys: M = 0.97, SD = 51, girls: M = 0.84, SD = 0.49, *F*(1,324) = 5.24, *p* < 0.05), CRAE (boys: M = 202.47 mu, SD = 13.32, girls: M = 207.71 mu, SD = 13.90, *F*(1,324) = 11.96, *p* < 0.001), CRVE (boys: M = 228.96 mu, SD = 11.80, girls: M = 233.52 mu, SD = 13.78, *F*(1,324) = 10.22, *p* < 0.01), and 20 m shuttle run performance (boys: M = 4.94 stages, SD = 1.70, girls: M = 4.20 stages, SD = 1.50, *F*(1,324) = 17.13, *p* < 0.001). Age was significantly correlated with performance on the shuttle run test (*r* = 0.15, *p* < 0.01), whereas parental/guardian education was significantly associated with children's family stress (*r* = 0.13, *p* < 0.05), peer stress (*r* = 0.13, *p* < 0.05), school stress (*r* = −0.27, *p* < 0.001), SBP (*r* = −0.11, *p* < 0.05), and physical fitness (*r* = 0.29, *p* < 0.001). Increased school stress was associated with higher BMI scores (*r* = 0.20, *p* < 0.001). Moreover, children with higher BMI scores had increased systolic (*r* = 0.31, *p* < 0.001) and diastolic blood pressure (*r* = 0.16, *p* < 0.01), whereas they performed less well in the 20 m shuttle run test (*r* = −0.33, *p* < 0.001).

### 3.2. Bivariate Associations between the Study Variables

Table 1 displays the bivariate correlations between all study variables. Small but significant (positive) associations were found between critical life events, family stress, and peer stress (*r*s between 0.13 and 0.25, *p* < 0.05). Similarly, peer stress was positively associated with school stress (*r* = 0.22, *p* < 0.001). Small inverse correlations were found between peer stress and SBP and DBP (*r* = −0.12 to −0.13, *p* < 0.05), as well as school stress and performance in the 20 m shuttle run test (*r* = −0.17, *p* < 0.01). A strong positive correlation existed between SBP and DBP (*r* = 0.70, *p* < 0.001), whereas small inverse relationships were found for SBP and DBP with CRAE and AVR (*r* = −0.23 to −0.26, *p* < 0.001). Moreover, higher CRAE scores were related to wider CRVE (*r* = 0.59, *p* < 0.001) and a higher AVR (*r* = 0.62, *p* < 0.001). By contrast, CRVE and AVR were negatively correlated with each other (*r* = −0.27, *p* < 0.001). No significant association occurred between performance on the 20 m shuttle run test and CRAE. However, small but significant relationships were found between physical fitness and both CRVE (*r* = −0.15, *p* < 0.01) and AVR (*r* = 0.12, *p* < 0.05).

### 3.3. Physical Fitness as a Buffer of Psychological Stress

As shown in Table 2, critical life events and 20 m shuttle run only explained minor amounts of variance in the five outcome variables, after controlling for sex, age, parental education, and BMI, while females had higher values for CRAE and DBP (*β* = 0.11, *p* < 0.05).

Higher BMI was associated with increased SBP (*β* = 0.32, *p* < 0.05) and DBP (*β* = 0.16, *p* < 0.05). Whereas a weak positive association was found between children's 20 m shuttle run performance and AVR (*β* = 0.13, *p* < 0.05), performance in the 20 m shuttle run test was negatively associated with CRVE (*β* = −0.12, *p* < 0.05). None of the two-way interactions between critical life events and physical fitness were significant. The only strong predictor was CRVE in the model with CRAE as the outcome (*β* = 0.57, *p* < 0.001) and vice versa.

With family stress as the predictor variable, Table 2 corroborates that only limited amounts of variance were explained by sex, age, parental education, BMI, family stress, and 20 m shuttle run. Beyond the findings described above, no significant main or interaction effects were identified.

**Figure 1:**
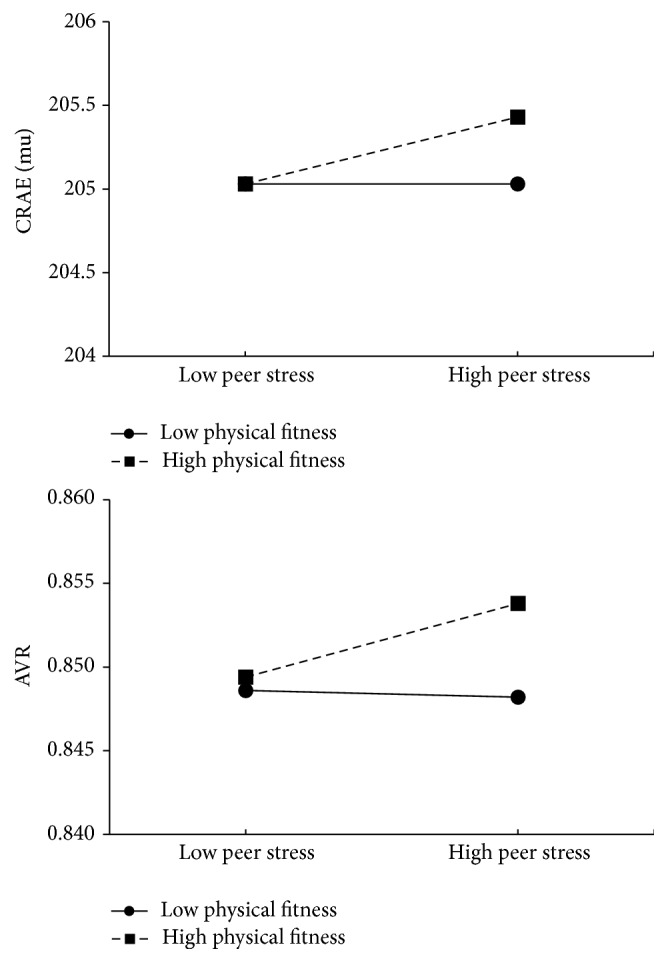
Interaction between peer stress and physical fitness on arteriolar vessel diameters (CRAE) and arteriolar to venular ratio (AVR), after controlling for gender, age, parental education, and BMI.

The level of explained variance for the final model was comparable if peer stress was used as a predictor variable (see Table 2). In contrast to the previous analyses, two significant interaction effects occurred for CRAE (*β* = 0.10, *p* < 0.05) and AVR (*β* = 0.12, *p* < 0.05).  Figure 1 illustrates that, among schoolchildren with low peer stress, physical fitness was not associated with CRAE, whereas among students with higher peer stress those with higher fitness levels had wider arteriolar diameters. A similar pattern was found for AVR, showing that the AVR was similar among students with low physical fitness if they experienced low peer stress, whereas schoolchildren with higher fitness had elevated AVR if they had high levels of peer stress.

**Figure 2:**
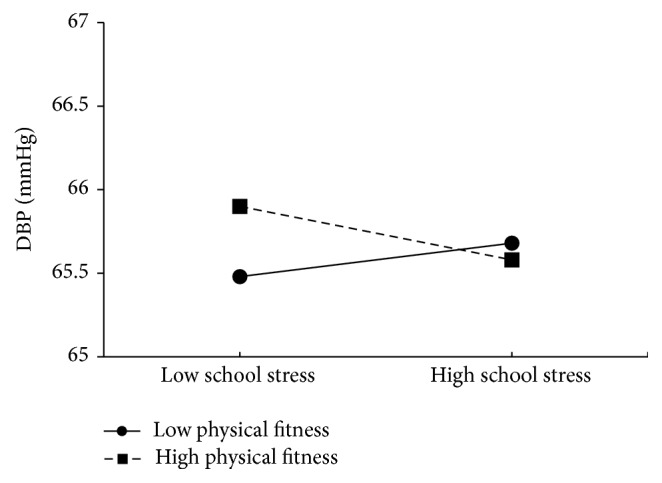
Interaction between school stress and physical fitness on diastolic blood pressure (DBP), after controlling for gender, age, parental education, and BMI.

With regard to school-related stress, one significant interaction effect was found for DBP (*β* = −0.13, *p* < 0.05). If DBP was used as outcome, the pattern of the findings suggests that, among students with low school stress, those children with low fitness levels had slightly lower blood pressure than peers with higher fitness levels, whereas among peers with increased school stress no substantial difference existed between children with low versus high fitness levels (Figure 2).

## 4. Discussion

In many respects, this study explored unknown territory. To the best of our knowledge, no research exists on how psychological stress is associated with retinal vessel diameters. Moreover, this is the first study to explore the potential stress-buffering effects associated with increased levels of physical fitness in primary schoolchildren. Finally, this study provides new insights into whether physical fitness moderates the relationship between psychological stress and cardiovascular disease risk factors among young schoolchildren.

The key findings of the present study are that critical life events, family, peer, and school-related stress are only weakly associated with retinal vessel diameters and blood pressure among first grade schoolchildren. Furthermore, the present findings do not support a stress-buffering effect of physical fitness in the stricter sense. Thus, whereas we found significant two-way interaction effects in three of 20 regression analyses, these showed (i) that students with high peer stress and high fitness levels had increased CRAE and AVR and (ii) that students with low school stress and high fitness levels had higher diastolic blood pressure, compared to peers with high stress and low physical fitness levels.

Thus, while the present investigation laid important groundwork, which can stimulate further research in children and adults, the findings raise more questions than they provide answers. The first relevant question is why no consistent associations were observed between children's psychological stress, retinal vessel diameters, and blood pressure? Based on adult research [57], we expected that elevated stress would be associated with less favourable cardiovascular disease risk profiles. Researchers have proposed several mechanisms to explain the way in which psychosocial stress affects cardiovascular health, including via a blunted baroreflex sensitivity, an elevation of arterial blood pressure, and increased neurohumoral arousal, glycated haemoglobin, lipid responses, and plasma fibrinogen responses to acute mental stress [58, 59]. The latter finding is in line with a meta-analysis showing that increased cardiovascular responses (e.g., heart rate and blood pressure) to experimentally induced stress are associated with higher risks of future hypertension, subclinical atherosclerosis, and cardiovascular disease events [60]. Furthermore, significant positive relationships were found in adults between psychological stress and indicators of arteriolar stiffness [61], which in turn was associated with retinal arteriolar narrowing [62]. In contrast to these findings, no significant associations were observed between psychosocial stress and retinal vessel diameters in our population, and correlations between stress and blood pressure were weak. These findings could be explained in a number of ways: first, children might not have been exposed to the assessed stressors for a sufficiently long time to display manifest cardiovascular disease risk factors. Thus, while psychosocial stress might be associated with indicators of mental ill-health early in life [63], it may take more time for psychosocial stress to impact cardiovascular disease risk factors. Second, the ability of parents/guardians to reliably report their children's stress levels might be questionable [46]. A previous study indicated that parents tend to underestimate the level of stress experienced by their offspring [64]. Nevertheless, few alternatives exist. For instance, the usefulness of objective stress biomarkers (such as hair cortisol concentrations) is still a matter of debate among stress researchers [65], and generally only weak relationships are observed between hair cortisol, life events, and subjective stress perceptions [66, 67]. Third, the schoolchildren in the present sample were comparably healthy; a previous study with the present population showed that only 14% were classified as hypertensive [68]. Hence, perceived stress might have been more closely associated with cardiovascular outcomes if more students had had clinically salient cardiovascular disease risk factors.

The second pressing question is why children with high physical fitness had increased arteriolar vessel diameters and a higher arteriolar to venular ratio if they were exposed to high peer stress. This finding was unexpected. Because psychosocial stress and exercise elicit similar central and peripheral responses and both cause substantial cardiovascular responses [5], it can be assumed that exposure to exercise-based stress would lead to an adaptation, which results in a blunted physiological response when individuals face psychosocial stress. A meta-analysis supports the notion that a single bout of acute exercise before a psychosocial stressor is associated with a reduced cardiovascular response compared to sedentary activities [69]. In addition, studies with adults have found less severe cardiovascular, endocrine, and inflammatory responses to psychosocial stress among physically fit adults compared to their unfit peers [70, 71], a finding which was supported in 8-year-old children [72]. Contrary to the expectations formulated based on these studies, however, no evidence for a standard stress-buffering effect was found in the present sample. The lack of significant two-way interactions in most of the regression analyses can be explained by the fact that only few significant associations occurred between psychosocial stress and retinal vessel diameters and blood pressure. Thus, the prerequisite for physical fitness to moderate the (expected) deleterious relationship between psychosocial stress and cardiovascular disease risk factors was not present [73]. Although speculative, the finding that increased arteriolar diameters and AVR scores were observed in children who simultaneously had high physical fitness and high stress levels may be explained by the fact that both psychological stress [74] and exercise [75] are associated with increased cardiac output. Thus, a cumulative effect might have resulted in wider arteries in this specific group of children. Nevertheless, we acknowledge that the precise physiological mechanisms behind these relationships remain unclear. We therefore suggest that more research is necessary to determine whether these results can be replicated in future investigations. Additionally, further research is needed with children to examine the potential of physical fitness as a stress-buffer by using more reliable cardiorespiratory fitness tests and other cardiovascular disease risk factors [76].

Despite these unexpected findings, some results correspond well with prior investigations. Higher CRAE and CRVE among girls have been observed previously in young children [37]. Moreover, significant associations between higher fitness, lower CRVE, and higher AVR have been identified before [27, 36, 37]. Finally, this study corroborates the evidence suggesting that close positive relationships exist between CRAE, CRVE, and AVR and that CRAE and AVR are negatively associated with systolic and diastolic blood pressure [26, 27, 33]. However, in contrast to previous research, only SBP and DBP (but not CRAE, CRVE, and AVR) were associated with BMI [30, 33].

The strengths of this study are that physical fitness was measured objectively with an established field-based test in a large sample of children of the same age [77]. Moreover, several validated instruments were used to assess children's levels of psychosocial stress [46], and all regression analyses were controlled for sociodemographic background and BMI before testing the main and interaction effects. Moreover, to consider refractive error, CRVE was included as a covariate in the models with CRAE as the outcome and CRAE in the models with CRVE as the outcome. Another strength of this study is the extensive imaging regime. Thus, a high degree of accuracy in the measurement of retinal vessel diameters was achieved by taking two retinal images of each eye per child.

Among the limitations of this study are the cross-sectional study design, which does not allow conclusions to be drawn about cause and effect. Since participation in the study was voluntary, a selection bias may have occurred. Furthermore, more research is needed to examine whether retinal arteriolar narrowing in young children really predicts cardiovascular outcome in adulthood or is simply a reflection of autoregulatory mechanisms in higher blood pressure among children. Moreover, due to the low age of the children, psychosocial stress was assessed by means of parental reports. Although no real alternatives exist, the question of the extent to which parents are able to validly gauge their children's stress perceptions cannot be overlooked [78]. Finally, the present study focused on two specific cardiovascular indicators, namely, retinal vessel diameters and blood pressure. Consequently, the degree to which physical fitness can buffer the negative impact of high stress on mental well-being remains unclear (e.g., depressive symptoms and self-esteem). While such information has been assessed in the present study, these data go beyond the scope of the present article and will be presented elsewhere.

## 5. Conclusions

On the basis of our results, we conclude that critical life events and more general psychological stress are not (or are, at best, weakly) associated with blood pressure and retinal vessel diameters. Support for a stress-buffering effect of high levels of physical fitness was not found in this sample of first grade primary schoolchildren. More research is needed with different age groups (including children, adolescents, adults, and the elderly) to find out if and from what age physical fitness can protect against arteriolar vessel narrowing and the occurrence of other cardiovascular disease risk factors.

## Figures and Tables

**Table 1:** Descriptive statistics and bivariate correlations between all study variables.

	M	SD	(1)	(2)	(3)	(4)	(5)	(6)	(7)	(8)	(9)	(10)
(1) Critical life events	1.54	2.58	—									
(2) Family stress	0.70	0.47	0.23^*∗∗∗*^	—								
(3) Peer stress	0.80	0.50	0.13^*∗*^	0.25^*∗∗∗*^	—							
(4) School stress	0.42	0.42	0.08	0.05	0.22^*∗∗∗*^	—						
(5) CRAE (mu)	205.13	13.85	−0.02	0.07	0.07	−0.01	—					
(6) CRVE (mu)	231.28	13.03	0.02	0.08	0.02	0.02	0.59^*∗∗∗*^	—				
(7) AVR	0.89	0.05	−0.04	0.00	0.07	−0.04	0.62^*∗∗∗*^	−0.27^*∗∗∗*^	—			
(8) Systolic blood pressure (mmHg)	104.62	8.03	−0.03	−0.08	−0.13^*∗*^	0.10	−0.25^*∗∗∗*^	−0.09	−0.23^*∗∗∗*^	—		
(9) Diastolic blood pressure (mmHg)	65.66	6.87	0.03	−0.01	−0.12^*∗*^	0.04	−0.26^*∗∗∗*^	−0.09	−0.23^*∗∗∗*^	0.70^*∗∗∗*^	—	
(10) 20 m shuttle run test (stages)	4.57	1.64	−0.07	0.07	−0.01	−0.17^*∗∗*^	−0.02	−0.15^*∗∗*^	0.12^*∗*^	−0.06	−0.01	—

Sex (0 = male, 1 = female)	—	—	0.00	0.03	−0.13^*∗*^	−0.08	0.19^*∗∗*^	0.18^*∗∗*^	0.06	0.00	0.10	−0.23^*∗∗∗*^
Age	7.28	0.36	−0.03	−0.03	0.02	−0.02	−0.11	−0.06	−0.07	0.09	0.09	0.15^*∗∗*^
Parental/guardian education	2.32	0.77	−0.04	0.13^*∗*^	0.13^*∗*^	−0.27^*∗∗∗*^	−0.01	−0.11	0.08	−0.09	−0.09	0.29^*∗∗∗*^
Body mass index (kg/m^2^)	16.33	2.03	0.06	−0.08	−0.06	0.20^*∗∗∗*^	−0.11	−0.04	−0.09	0.31^*∗∗∗*^	0.16^*∗∗*^	−0.33^*∗∗*^

*Note*. ^*∗*^ *p* < 0.05.^*∗∗*^ *p* < 0.01. ^*∗∗∗*^ *p* < 0.001.

**Table 2:** Regression analyses with critical life events, family stress, peer stress, and school stress as independent variables, fitness as a moderator, and retinal vessel diameters as outcomes.

	Critical life events		Family stress		Peer stress		School stress	
CRAE	Δ*R* ^2^	*β*	Δ*R* ^2^	*β*	Δ*R* ^2^	*β*	Δ*R* ^2^	*β*

Step 1:	0.365^*∗∗∗*^	—	0.365^*∗∗∗*^	—	0.365^*∗∗∗*^	—	0.365	—
Sex	—	0.11^*∗*^	—	0.11^*∗*^	—	0.12^*∗*^	—	0.11^*∗*^
Age	—	−0.08	—	−0.08	—	−0.08	—	−0.08
Parental education	—	0.01	—	0.01	—	−0.02	—	0.01
BMI	—	−0.05	—	−0.05	—	−0.04	—	−0.06
CRVE	—	0.57^*∗∗∗*^	—	0.57^*∗∗∗*^	—	0.57^*∗∗∗*^	—	0.57^*∗∗∗*^
Step 2: stress	0.000	−0.02	0.000	0.01	0.005	0.10^*∗*^	0.000	0.01
Step 3: fitness	0.005	0.08	0.005	0.08	0.006	0.10	0.005	0.08
Step 4: stress × fitness	0.000	−0.01	0.002	0.05	0.010^*∗*^	0.10^*∗*^	0.000	−0.02
Total *R* ^2^	0.370^*∗∗∗*^		0.372^*∗∗∗*^		0.386^*∗∗∗*^		0.370^*∗∗∗*^	
*n*	325		325		325		325	

CRVE	*ΔR* ^2^	*β*	*ΔR* ^2^	*β*	*ΔR* ^2^	*β*	*ΔR* ^2^	*β*

Step 1:	0.359^*∗∗∗*^	—	0.359^*∗∗∗*^	—	0.359^*∗∗∗*^	—	0.359^*∗∗∗*^	—
Sex	—	0.04	—	0.04	—	0.04	—	0.04
Age	—	0.01	—	0.02	—	0.02	—	0.02
Parental education	—	−0.07	—	−0.08	—	−0.07	—	−0.08
BMI	—	−0.04	—	−0.04	—	−0.04	—	−0.04
CRAE	—	0.57^*∗∗∗*^	—	0.57^*∗∗∗*^	—	0.58^*∗∗∗*^	—	0.57^*∗∗∗*^
Step 2: stress	0.001	0.02	0.003	0.06	0.000	0.03	0.000	−0.01
Step 3: fitness	0.011^*∗*^	−0.12^*∗*^	0.012^*∗*^	−0.13^*∗*^	0.011^*∗*^	−0.13^*∗*^	0.011^*∗*^	−0.12^*∗*^
Step 4: stress × fitness	0.000	−0.00	0.000	0.02	0.003	−0.05	0.001	−0.04
Total *R* ^2^	0.370^*∗∗∗*^		0.374^*∗∗∗*^		0.373^*∗∗∗*^		0.371^*∗∗∗*^	
*n*	325		325		325		325	

AVR	*ΔR* ^2^	*β*	*ΔR* ^2^	*β*	*ΔR* ^2^	*β*	*ΔR* ^2^	*β*

Step 1:	0.018	—	0.018	—	0.018	—	0.018	—
Sex	—	0.09	—	0.09	—	0.09	—	0.09
Age	—	−0.08	—	−0.09	—	−0.09	—	−0.08
Parental education	—	0.03	—	0.03	—	0.02	—	0.03
BMI	—	−0.03	—	−0.03	—	−0.02	—	−0.03
Step 2: stress	0.001	−0.03	0.000	−0.02	0.004	0.10	0.000	0.01
Step 3: fitness	0.013^*∗*^	0.13^*∗*^	0.013^*∗*^	0.13^*∗*^	0.015^*∗*^	0.15^*∗*^	0.013^*∗*^	0.13^*∗*^
Step 4: stress × fitness	0.000	−0.01	0.001	0.04	0.014^*∗*^	0.12^*∗*^	0.000	0.01
Total *R* ^2^	0.032		0.032		0.052^*∗*^		0.032	
*n*	325		325		325		325	

Systolic blood pressure	*ΔR* ^2^	*β*	*ΔR* ^2^	*β*	*ΔR* ^2^	*β*	*ΔR* ^2^	*β*

Step 1:	0.102^*∗∗∗*^		0.102^*∗∗∗*^		0.102^*∗∗∗*^		0.102^*∗∗∗*^	
Sex	—	−0.01	—	0.00	—	−0.02	—	0.00
Age	—	0.10	—	0.04	—	0.05	—	0.05
Parental education	—	−0.09	—	−0.05	—	−0.04	—	−0.05
BMI	—	0.32^*∗∗∗*^	—	0.31^*∗∗∗*^	—	0.30^*∗∗∗*^	—	0.29^*∗∗∗*^
Step 2: stress	0.002	−0.09	0.002	−0.06	0.013^*∗*^	−0.12^*∗*^	0.001	0.01
Step 3: fitness	0.002	−0.06	0.003	−0.06	0.001	−0.04	0.003	−0.05
Step 4: stress × fitness	0.009	−0.10	0.002	−0.05	0.003	−0.06	0.006	−0.08
Total *R* ^2^	0.115^*∗∗∗*^		0.109^*∗∗∗*^		0.119^*∗∗∗*^		0.112^*∗∗∗*^	
*n*	325		325		325		325	

Diastolic blood pressure	*ΔR* ^2^	*β*	*ΔR* ^2^	*β*	*ΔR* ^2^	*β*	*ΔR* ^2^	*β*

Step 1:	0.045^*∗∗*^		0.045^*∗∗*^		0.045^*∗∗*^		0.045^*∗∗*^	
Sex	—	0.11^*∗*^	—	0.12^*∗*^	—	0.11^*∗*^	—	0.11^*∗*^
Age	—	0.07	—	0.06	—	0.06	—	0.07
Parental education	—	−0.07	—	−0.08	—	−0.06	—	−0.08
BMI	—	0.16^*∗∗*^	—	0.16^*∗∗*^	—	0.14^*∗*^	—	0.14^*∗*^
Step 2: stress	0.000	−0.01	0.000	−0.00	0.010	−0.11^*∗*^	0.000	−0.03
Step 3: fitness	0.005	0.09	0.005	0.09	0.004	0.06	0.005	0.08
Step 4: stress × fitness	0.004	−0.07	0.007	−0.08	0.008	−0.09	0.015^*∗∗*^	−0.13^*∗∗*^
Total *R* ^2^	0.055^*∗*^		0.057^*∗*^		0.067^*∗∗*^		0.065^*∗∗∗*^	
*n*	325		325		325		325	

*Note.* All analyses controlled for sex, age, parental education, and BMI. CRAE was controlled in the models with CRVE as the outcome and CRVE in the models with CRAE as the outcome. ^*∗*^ *p* < 0.05. ^*∗∗*^ *p* < 0.01. ^*∗∗∗*^ *p* < 0.001.
